# 17q12 Microdeletion Syndrome Initially Presenting with Tremor in Maturity-Onset Diabetes of the Young

**DOI:** 10.1007/s12098-025-05627-2

**Published:** 2025-06-21

**Authors:** Xuewen Yuan, Wei Gu

**Affiliations:** Department of Endocrinology, Genetics and Metabolism, Children’s Hospital of Nanjing Medical University, Nanjing, 210000 Jiangsu Province China

*To the Editor*: A 12-y-old boy presented to the hospital with unexplained tremor of the upper limbs for a week. Further laboratory tests showed a magnesium level of 0.50 mmol/L, fasting blood glucose of 15.48 mmol/L, abnormal liver function, and significantly reduced insulin and C-peptide. A complete CT and MRI examination showed partial atrophy of the pancreas and full left renal pelvis and bilateral lateral ventricles. Further genetic testing revealed a 1.84 Mb copy number deletion in the 17q12 region of the long arm of chromosome 17 (covering HNF1B, ZNHIT3 and other genes), which was classified to be pathogenic. Combined with diabetes, hypomagnesemia, liver dysfunction and pancreatic atrophy, the patient was diagnosed with 17q12 microdeletion syndrome complicated with Maturity Onset Diabetes of the Young type 5 (MODY5) [1]. During hospitalization, the blood glucose and tremor symptoms of the child were closely monitored. During the follow-ups, the patient was in good health, and the blood glucose and liver functions recovered well.

Rare mutations in the hepatocyte nuclear factor 1-β (HNF1B) gene produce a syndrome that resembles MODY and has been termed MODY5 [2]. Approximately 50% of MODY5 patients harbor a whole gene deletion, which is associated with severe pathology, hyperglycemia, and progressive pancreatic beta-cell dysfunction, known as 17q12 microdeletion syndrome [3]. It is typically characterized by the deletion of some genes, leading to renal abnormalities, renal cysts and diabetic syndromes with neurodevelopmental or neuropsychiatric disorders [4].

This case report describes a rare child with 17q12 microdeletion syndrome combined with MODY5 type diabetes. Through comprehensive assessment of early clinical symptoms and genetic testing, an accurate diagnosis and timely treatment were made, effectively preventing severe complications. Comprehensive treatment, regular follow-ups, and personalized care adjustments will significantly enhance the quality of life and ensure long-term health for such children.
